# Tankyrase Regulates Neurite Outgrowth through Poly(ADP-ribosyl)ation-Dependent Activation of β-Catenin Signaling

**DOI:** 10.3390/ijms23052834

**Published:** 2022-03-04

**Authors:** Masato Mashimo, Momoko Kita, Arina Uno, Moe Nii, Moe Ishihara, Takuya Honda, Yuka Gotoh-Kinoshita, Atsuo Nomura, Hiroyuki Nakamura, Toshihiko Murayama, Ryoichi Kizu, Takeshi Fujii

**Affiliations:** 1 Department of Pharmacology, Faculty of Pharmaceutical Sciences, Doshisha Women’s College of Liberal Arts, Kyotanabe 610-0395, Japan; ykj045@dwc.doshisha.ac.jp (M.K.); dyl36.0228@gmail.com (A.U.); ykg071@dwc.doshisha.ac.jp (M.N.); ykk020@dwc.doshisha.ac.jp (M.I.); a-nomura@dwc.doshisha.ac.jp (A.N.); tfujii@dwc.doshisha.ac.jp (T.F.); 2 Laboratory of Chemical Pharmacology, Graduate School of Pharmaceutical Sciences, Chiba University, Chiba 260-8675, Japan; t-honda@chiba-u.jp (T.H.); nakahiro@faculty.chiba-u.jp (H.N.); murayama-toshi@faculty.chiba-u.jp (T.M.); 3 Department of Clinical Pharmacy, Faculty of Pharmaceutical Sciences, Doshisha Women’s College of Liberal Arts, Kyotanabe 610-0395, Japan; youjia.backwisteria@gmail.com (Y.G.-K.); rkizu@dwc.doshisha.ac.jp (R.K.)

**Keywords:** tankyrase, poly(ADP-ribosyl)ation, neurite outgrowth, β-catenin

## Abstract

Poly(ADP-ribosyl)ation is a post-translational modification of proteins by transferring poly(ADP-ribose) (PAR) to acceptor proteins by the action of poly(ADP-ribose) polymerase (PARP). Two tankyrase (TNKS) isoforms, TNK1 and TNK2 (TNKS1/2), are ubiquitously expressed in mammalian cells and participate in diverse cellular functions, including wnt/β-catenin signaling, telomere maintenance, glucose metabolism and mitosis regulation. For wnt/β-catenin signaling, TNKS1/2 catalyze poly(ADP-ribosyl)ation of Axin, a key component of the β-catenin degradation complex, which allows Axin’s ubiquitination and subsequent degradation, thereby activating β-catenin signaling. In the present study, we focused on the functions of TNKS1/2 in neuronal development. In primary hippocampal neurons, TNKS1/2 were detected in the soma and neurites, where they co-localized with PAR signals. Treatment with XAV939, a selective TNKS1/2 inhibitor, suppressed neurite outgrowth and synapse formation. In addition, XAV939 also suppressed norepinephrine uptake in PC12 cells, a rat pheochromocytoma cell line. These effects likely resulted from the inhibition of β-catenin signaling through the stabilization of Axin, which suggests TNKS1/2 enhance Axin degradation by modifying its poly(ADP-ribosyl)ation, thereby stabilizing wnt/β-catenin signaling and, in turn, promoting neurite outgrowth and synapse formation.

## 1. Introduction

Poly(ADP-ribose) polymerase (PARP) catalyzes ADP-ribosylation, a reversible post-translational protein modification in which one or more ADP-ribose units are transferred from donor NAD^+^ to target proteins [1,2,3]. This modification alters the activity and subcellular localization of the target proteins, thereby regulating diverse cellular functions. The PARP family consists of 17 members that differ in their localization and enzymatic activity. Among them, tankyrase 1 and 2 (TNKS1/2), also known as PARP5a and PARP5b, respectively, each contain two unique structural domains not contained by other PARP members: an ankyrin repeat domain and a sterile alpha motif (SAM). The ankyrin repeat domain serves as the interface for interaction with acceptor proteins, while the SAM regulates the polymerization of TNKS [4,5,6,7]. As TNKS1 and 2 are highly homologous and exhibit similar localizations at telomers, centrosomes, nuclear pores, Golgi complexes, cytoplasm and peroxisomes, their functions appear to overlap. Consistent with that idea, deletion of the gene encoding either TNKS1 or TNKS2 does not result in significant changes in mice, but their double knockout is embryonically lethal [8,9]. As implied by the lethality of TNKS1/2 double deletion, TNKS1/2-catalyzed poly(ADP-ribosyl)ation is involved in a variety of essential physiological processes. For example, knockdown of TNKS1 results in mitotic arrest, suggesting that TNKS1 is required for sister chromatid resolution, which is necessary for mitotic progression [10,11].

TNKS1/2 are also known to regulate the wnt/β-catenin pathway involved in cell proliferation and differentiation [12,13,14]. The cellular level of β-catenin is controlled by the β-catenin destruction complex composed of Axin, adenomatosis polyposis coli (APC), protein phosphatase 2A (PP2A), glycogen synthase kinase 3 (GSK3) and casein kinase 1α (CK1α) [15,16]. Two serine residues on β-catenin are phosphorylated by GSK3β, which allows its ubiquitination by TrCP1, an E3 ubiquitin ligase, and its subsequent proteasomal degradation [17,18]. TNKS1/2 catalyze poly(ADP-ribosyl)ation of Axin, enabling its ubiquitination by RNF146, an E3 ubiquitin-protein ligase, and proteasomal degradation [13,19]. Axin degradation disrupts the β-catenin destruction complex, after which the stabilized β-catenin is translocated to the nucleus, where it binds to TCF/LEF, which mediates the transcription of several genes.

TNKS1/2 are ubiquitously expressed in mammalian cells, especially in the brain, ganglia, skin, and heart. However, the function of TNKS1/2 in the nervous system remains unclear due in large part to the embryonic lethality of TNKS1/2 double deletion. In the present study, however, we investigated the effect of TNKS1/2 on neurite outgrowth in mouse primary hippocampal neurons and rat adrenal medullary neuron-like cells, PC12 cells.

## 2. Results

### 2.1. TNKS1/2-Mediated Neurite Outgrowth and Synapse Formation in Primary Hippocampal Neurons

**Figure 1:**
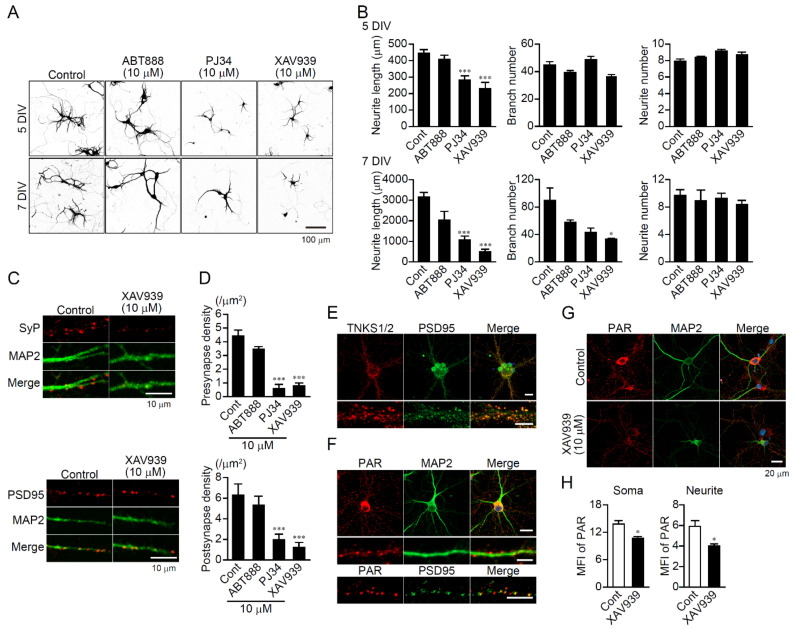
XAV939 prevents neurite outgrowth and synapse formation in primary hippocampal neurons. (**A**) Effect of PARP inhibitors on neurite outgrowth in primary hippocampal neurons. Neurons were treated with PARP inhibitors (10 μM) after 1 day in vitro (DIV) for 5 or 7 DIV. The neurites were immunostained with anti-MAP2 antibody. Scale bar: 100 μm. (**B**) Mean neurite length, branch number, and neurite number sprouting from the soma in primary hippocampal neurons after 5 or 7 DIV. The length and number of neurites stained with anti-MAP2 antibody was measured using NeurophologyJ. Data are shown as means ± SEM (*n* = 930–3406 cells) * *p* < 0.05, *** *p* < 0.001 vs. Control (one-way ANOVA with post hoc Dunnett’s test). (**C**) Effect of XAV939 on synapse formation. Neurons were treated with XAV939 (10 μM) after 1 DIV. After 5 DIV, cells were immunostained with anti-MAP2 (green) and anti-syptophysin (SyP) (red, left) or anti-PSD-95 (red, right) antibodies. Scale bar: 10 μm. (**D**) Effect of PARP inhibitors on pre- and postsynaptic density in neurites. PARP inhibitors (10 μM) were added for 5 DIV after 1 DIV. Numbers of SyP and PSD-95 punctations on neurites per 1 μm^2^ were calculated. Data are shown as means ± SEM (*n* = 4 images) *** *p* ˂ 0.001 (one-way post hoc Dunnett’s test). There are about 10 neurons per image. (**E**) Subcellular localization of TNKS1/2 in neurons. Primary hippocampal neurons (14 DIV) were immunostained with anti-PAR (red) and anti-PSD-95 (green) antibodies. Lower panels show magnified images of neurites. DAPI (blue in merged image) was used as a nuclear marker. Scale bars: 20 μm (upper), 10 μm (lower). (**F**) Subcellular localization of PAR in neurons. Primary hippocampal neurons (5 DIV) were immunostained with anti-PAR (red) and anti-MAP2 (upper, green) or anti-PSD-95 (lower, green) antibodies. Lower panels show magnified images of neurites. DAPI (blue in merged image) was used as a nuclear marker. Scale bars: 20 μm (upper), 10 μm (middle and lower). (**G**) Effect of XAV939 on PAR production in neurons. After 24 h treatment with XAV939 (10 μM), primary hippocampal neurons (5 DIV) were immunostained with anti-PAR (red) and anti-MAP2 (green) antibodies. DAPI (blue in merged image) was used as a nuclear marker. Scale bar: 20 μm. (**H**) Mean fluorescence intensity of PAR in soma and neurites. Data are shown as means ± SEM (*n* = 4 images). Approximately 10 neurons are present in an image. * *p* < 0.05 (Student’s *t*-test).

To understand the role of PARP isoforms in neurons, beginning one day after the start of culture, primary hippocampal neurons were incubated with various PARP inhibitors, including ABT888, a selective PARP1 and PARP2 inhibitor; PJ34, a nonselective PARP inhibitor; and XAV939, a selective TNKS1/2 inhibitor. The neurites were stained with anti-MAP2 antibody to measure their length and number. PJ34 and XAV939, but not ABT888, suppressed neurite length significantly after 5 and 7 days in vitro (DIV), though they did not affect the number of neurites sprouting from the soma (Figure 1A,B). XAV939 also reduced the number of neurite branches significantly after 7 DIV (Figure 1B). In addition, PJ34 and XAV939, but not ABT888, suppressed the density of presynaptic staining with anti-synaptophysin antibody and postsynaptic staining with anti-PSD95 antibody on the anti-MAP2 antibody-stained neurites (Figure 1C,D). As XAV939 and PJ34 exert inhibitory effects on TNKS1/2, these results indicate that TNKS1/2, but not PARP1, are involved in neurite outgrowth and synapse formation, but not neurite sprouting.

TNKS1/2 were present in the soma and neurites of primary hippocampal neurons after 5 DIV (Figure 1E). The enzymes were distributed in a punctate pattern within neurites, where they co-localized with PSD-95, suggesting they localize to synapses (Figure 1E, lower). Consistent with the observed distribution of TNKS1/2, poly(ADP-ribose) (PAR) was detected in the soma and at synapses on neurites (Figure 1F). Treatment with XAV939 for 24 h suppressed the PAR levels seen in those regions (Figure 1G,H), which suggests TNKS1/2 localize to the soma and neurites, where they catalyze poly(ADP-ribosyl)ation.

### 2.2. TNKS1/2 Participate in Neurite Outgrowth in PC12 Cells

**Figure 2:**
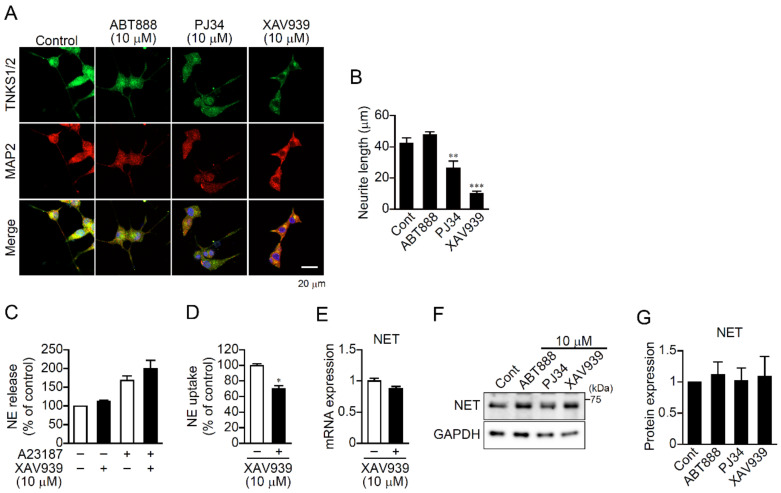
XAV939 prevents neurite outgrowth and NE uptake in PC 12 cells. (**A**) Effect of PARP inhibitors on neurite outgrowth in PC12 cells. Cells were treated with the indicated PARP inhibitor (10 μM) for 4 days in the presence of NGF (100 ng/mL). PC12 cells were immunostained with anti-TNKS1/2 (green in merged image) and anti-MAP2 (red in merged image) antibodies. DAPI (blue in merged image) was used as a nuclear marker. Scale bar: 20 μm. (**B**) Mean neurite length. Lengths of neurites stained with anti-MAP2 antibody were analyzed. Data are shown as means ± SEM (*n* = 14–18 cells) ** *p* < 0.01, *** *p* < 0.001 vs. Control (one-way ANOVA with post hoc Dunnett’s test). (**C**) Effect of XAV939 on NE release. PC12 cells was cultured with [^3^H]-NE for 1 day in the absence or presence of XAV939 (10 μM). [^3^H]-NE levels in the media were measured with a scintillation counter. A23187 was used to enhance Ca^2+^-dependent NE release. Data are shown as means ± SEM (*n* = 3). (**D**) Effect of XAV939 on NE uptake. PC12 cells was cultured with [^3^H]-NE for 18 h in the absence or presence of XAV939 (10 μM). Cellular [^3^H]-NE content was measured with a scintillation counter. Data are shown as means ± SEM (*n* = 3) * *p* < 0.05 vs. Control (Student’s *t*-test). (**E**) Effect of XAV939 on NET mRNA levels. After treatment with XAV939 (10 μM) for 24 h, PC12 cells were subjected to real-time PCR with NET primers. GAPDH was used as a loading control. Data are shown as means ± SEM (*n* = 3). (**F**) Effect of PARP inhibitors on NET protein expression. After treatment with the indicated inhibitor (10 μM) for 24 h, PC12 cells were subjected to Western blotting with an anti-NET antibody. GAPDH was used as a loading control. Data are shown as means ± SEM (*n* = 3). (**G**) Effect of PARP inhibitors on the relative levels of NET protein. Data are shown as means ± SEM (*n* = 3).

To investigate the intracellular mechanism by which TNKS1/2 regulate neurite outgrowth in neurons, PC12 rat pheochromocytoma cells were used as a model. After 4 days of nerve growth factor (NGF) treatment in low-serum medium, neurite outgrowth was observed (Figure 2A). As in hippocampal neurons, TNKS1/2 were localized in cell bodies and neurites (Figure 2A), and PJ34 and XAV939, but not ABT888, which suppressed MAP2-positive neurite outgrowth (Figure 2A,B). This suggests TNKS1/2 participate in neurite outgrowth in PC12 cells as they do in primary hippocampal neurons.

PC12 cells synthesize and release catecholamines and then take them up [20]. To assess the effect of XAV939 on those actions, PC12 cells were incubated with [^3^H]-labeled norepinephrine (NE), after which the [^3^H]-NE content of the cells and its secretion were measured. Interestingly, XAV939 had no effect on Ca^2+^-dependent or -independent [^3^H]-NE release (Figure 2C), but markedly inhibited [^3^H]-NE uptake (Figure 2D). NE transporter (NET) mediates NE uptake into cells, and Western blotting and real-time PCR revealed that XAV939 did not alter levels of NET protein or mRNA (Figure 2E–G). Apparently, XAV939 inhibits NE uptake by suppressing NET activity but not its expression.

### 2.3. TNKS1/2 Inhibition Upregulates Its Expression

**Figure 3:**
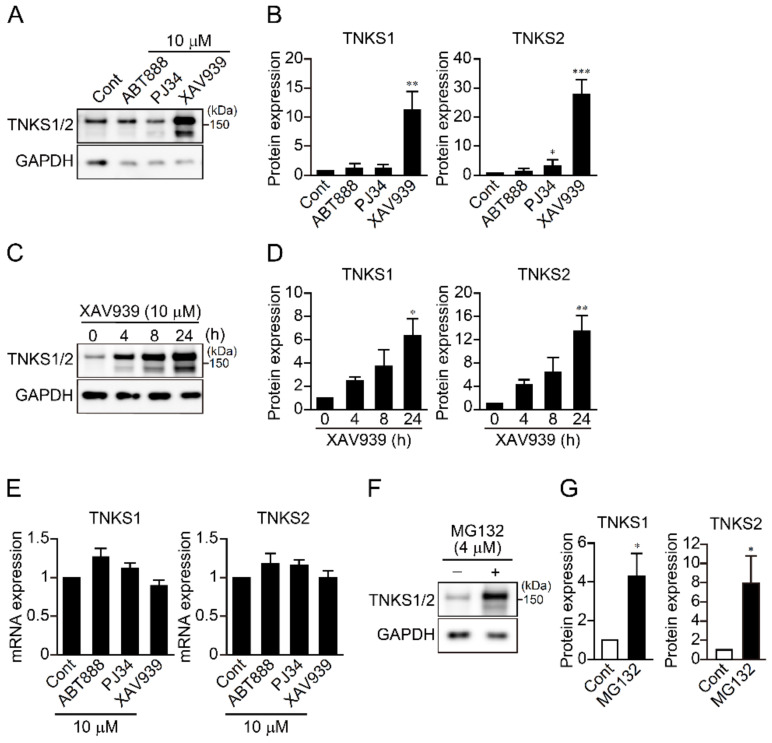
XAV939 upregulates TNKS1/2 expression. (**A**) Effect of PARP inhibitors on TNKS1/2 expression in PC12 cells. Cells were treated for 24 h with the indicated PARP inhibitor (10 μM) and then subjected to Western blotting with an anti-TNKS1/2 antibody. GAPDH was used as a loading control. (**B**) Relative levels of TNKS1/2 proteins after treatment for 24 h with the indicated PARP inhibitor. Data are shown as means ± SEM (*n* = 3). * *p* < 0.05, ** *p* < 0.01, *** *p* < 0.001 vs. Control (one-way ANOVA with post hoc Dunnett’s test). (**C**) Time course of TNKS1/2 protein expression in PC12 cells after treatment with XAV939 (10 μM) for the indicated times. (**D**) Relative TNKS1/2 protein expression after treatment with XAV939 for indicated times. Data are shown as means ± SEM (*n* = 3). * *p* < 0.05, ** *p* < 0.01 vs. Control (one-way ANOVA with post hoc Dunnett’s test). (**E**) Effect of PARP inhibitors on TNKS1/2 mRNA expression. After treatment for 24 h with the indicated PARP inhibitors (10 μM), PC12 cells were subjected to real-time PCR with TNKS1/2 primers. GAPDH was used as a loading control. Data are shown as means ± SEM (*n* = 3). (**F**) Effect of proteasome inhibition on TNKS1/2 protein levels. Cells were treated with MG132 (4 μM) for 24 h, followed by Western blotting using an anti-TNKS1/2 antibody. GAPDH was used as a loading control. (**G**) Relative TNKS1/2 protein levels after treatment for 24 h with MNG132. Data are shown as means ± SEM (*n* = 3). * *p* < 0.05 vs. Control (Student’s *t*-test).

We next examined the effects of PARP inhibitors on expression of TNKS1/2 protein. We found that XAV939 markedly increased levels of TNKS1/2, while PJ34 increased only TNKS2 and ABT888 had no effect (Figure 3A,B). XAV939 increased levels of TNKS1/2 over time so that their highest levels were detected after 24 h, the last measurement made (Figure 3C,D). Given that none of the PARP inhibitors affected TNKS1/2 mRNA expression (Figure 3E), it appears that PARP inhibitors do not affect TNKS1/2 transcription. In addition, the proteasome inhibitor MG 132 also increased TNKS1/2 levels in PC12 cells (Figure 3F,G), which suggests TNKS1/2 enzyme activity is likely to be involved in its own degradation by the proteasome. 

### 2.4. β-Catenin Pathway Mediates Neurite Outgrowth in PC12 Cells

**Figure 4:**
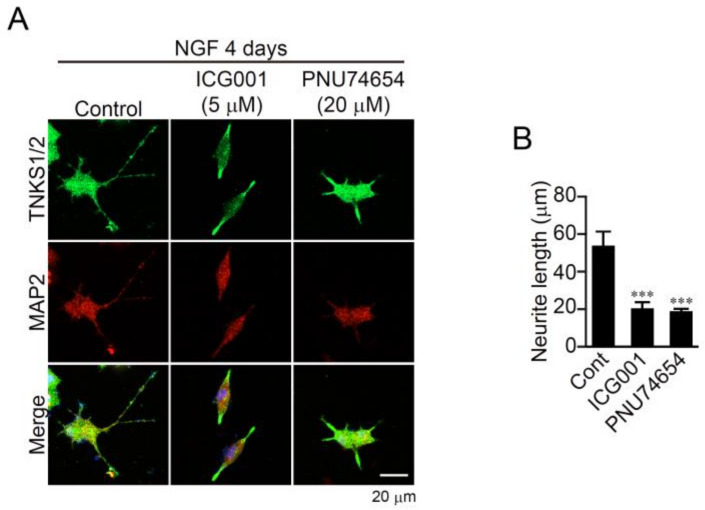
β-catenin pathway mediates neurite outgrowth in PC12 cells. (**A**) Effect of β-catenin inhibitors on TNKS1/2 localization and neurite outgrowth in PC12 cells. Cells were treated with ICG-001 (5 μM) and PNU-74654 (20 μM) after 1 day in culture with NGF (100 ng/mL). After 4 days, cells were stained with anti-TNKS1/2 and anti-MAP2 antibodies. Nuclei were stained with DAPI. Scale bar: 20 μm. (**B**) Mean neurite length in PC12 cells. Data are shown as means ± SEM (*n* = 14–18). *** *p* < 0.001 vs. Control (one-way ANOVA with post hoc Dunnett’s test).

TNKS1/2 reportedly regulate the wnt/β-catenin pathway [12,13,19]. To determine whether XAV939 inhibits neurite outgrowth by suppressing β-catenin signaling, we first assessed the involvement of β-catenin signaling in neurite outgrowth. The β-catenin pathway inhibitors ICG001 and PNU74654 both suppressed NGF-induced neurite outgrowth in PC12 cells (Figure 4A,B), confirming the involvement of the β-catenin pathway in neurite outgrowth. 

### 2.5. TNKS1/2 Regulate β-Catenin Signaling through Poly(ADP-ribosyl)ation

**Figure 5:**
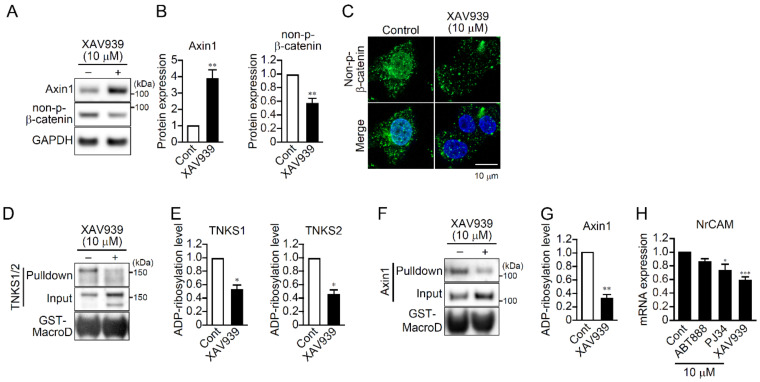
TNKS1/2 regulate β-catenin signaling through poly(ADP-ribosyl)ation in PC12 cells. (**A**) Effect of XAV939 on protein expression of Axin1 and unphosphorylated (non-*p*)-β-catenin in PC12 cells. Cells were treated with XAV939 (10 μM) for 1 day, followed by Western blotting with the indicated antibodies. GAPDH was used as a loading control. (**B**) Relative protein expression of Axin1 and non-p-β-catenin. Data are shown as means ± SEM (*n* = 3). ** *p* < 0.01 vs. Control (Student’s *t*-test). (**C**) Effect of XAV939 on subcellular localization of non-p-β-catenin in PC12 cells. Cells were treated for 1 day with XAV939 (10 μM) and then stained using an anti-non-p-β-catenin antibody. Nuclei were stained with DAPI. Scale bar: 20 μm. (**D**) and (**F**) Effect of XAV939 on poly(ADP-ribosyl)ation of TNKS1/2 (**D**) and Axin1 (**F**) in PC12 cells. Cells were treated for 4 h with XAV939 (10 μM) and then subjected to macrodomain (MacroD)-GST pulldown assays followed by Western blotting with the indicated antibodies. (**E**) and (**G**) Relative poly(ADP-ribosyl)ation levels on TNKS1/2 (**E**) and Axin1 (**G**). Data are shown as means ± SEM (*n* = 4). * *p* < 0.05, *** *p* < 0.001 vs. Control (Student’s *t*-test). (**H**) Effect of PARP inhibitors on NrCAM mRNA expression in PC12 cells. Cells were treated for 24 h with the indicated PARP inhibitors (10 μM) and then subjected to real-time PCR using NrCAM primers. NrCAM mRNA levels were normalized to GAPDH mRNA. Data are shown as means ± SEM (*n* = 3) * *p* < 0.05, *** *p* < 0.001 vs. Control (one-way ANOVA with post hoc Dunnett’s test).

TNKS1/2 activate β-catenin signaling through poly(ADP-ribosyl)ation of Axin, which leads to its ubiquitination and degradation [12,13,19]. In PC12 cells, XAV939 significantly increased Axin1 levels and decreased levels of unphosphorylated (activated) β-catenin (Figure 5A,B). In addition, XAV939 suppressed localization of unphosphorylated β-catenin within the nucleus (Figure 5C). Macrodomain GST pulldown assays to isolate ADP-ribosylated proteins showed that ADP-ribosylation of TNKS1/2 and Axin1 was suppressed by XAV939 (Figure 5D–G). These results confirm that TNKS1/2 catalyze poly(ADP-ribosyl)ation of Axin1, thereby promoting β-catenin signaling.

After translocation to the nucleus, β-catenin binds to the transcription factors TCF/LEF and promotes gene expression [15,16]. NrCAM is a neural cell adhesion molecule involved in neurite outgrowth, and its expression is upregulated via the wnt/β-catenin pathway [21,22,23]. XAV939 and PJ34, but notABT888, decreased expression of NrCAM mRNA (Figure 5H), suggesting TNKS1/2 promote both wnt/β-catenin signaling and NrCAM transcription.

## 3. Discussion

In this study, we found that TNKS1/2 are present on the cell bodies and neurites of primary hippocampal neurons and participate in neurite elongation and synapse formation. Using PC12 cells to investigate the mechanism by which TNKS1/2 affect neurite outgrowth, it was found that activation of wnt/β-catenin signaling through poly(ADP-ribosyl)ation-mediated degradation of Axin1 is a key factor.

The PARP family has 17 members with different activities and subcellular localizations and specific biological activities associated with both normal physiological and pathophysiological processes [1,2,3]. Indeed, inhibition of PARP activity has clinical benefits in several diseases [24,25,26,27,28]. PARP inhibitors have been developed based on the unique structural features of each isoform, especially structural differences in the NAD^+^-binding domain [28,29]. Several of these inhibitors are currently in clinical trials or are now being used in clinical practice [25,30,31,32]. In particular, PARP1- and PARP2-specific inhibitors are being used in combination with conventional anticancer drugs to promote synthetic lethality in cancer cells with BRCA1/2 mutations [33,34]. The PARP inhibitors used in the present study (PJ34, ABT888, and XAV939) have differing inhibition profiles against PARP members: ABT888 is 1000-fold more specific for PARP1/2 than TNKS1/2, while XAV939 is 200-fold more specific for TNKS1/2 than for PARP1 [28,29]. PJ34 is relatively nonspecific but shows slightly stronger inhibition against PARP1 and PARP2. Among these three inhibitors, XAV939 exhibits by far the greatest ability to inhibit elongation of hippocampal neurons and PC12 cells, as their relative efficacies are XAV939 > PJ34 > > > ABT-888. This suggests TNKS1/2, but not PARP1/2, are involved in neurite outgrowth, which is consistent with the earlier finding that mice lacking PARP1 grow normally and show no abnormalities in brain development [35].

It has been reported that wnt/β-catenin signaling contributes to the regulation of stem cell pluripotency and cell fate during development in addition to neuronal axon guidance and synapse formation [16,34,36]. As mentioned, β-catenin is phosphorylated by the β-catenin destruction complex consisting of APC, Axin1 and GSK3β, which is maintained at low levels through targeted ubiquitination and proteasomal degradation [4,15,16]. Once TNKS1/2 catalyze poly(ADP-ribosyl)ation of Axin1, it is recognized by the E3 ubiquitin ligase RNF146, which contains a PAR-binding WWE domain, leading to ubiquitination-dependent degradation [13,19]. In the present study, XAV939 suppressed poly(ADP-ribosyl)ation of Axin1 in PC12 cells. The resultant stabilization of Axin1 led to decreased levels of active unphosphorylated β-catenin and suppression of its nuclear translocation.

**Figure 6:**
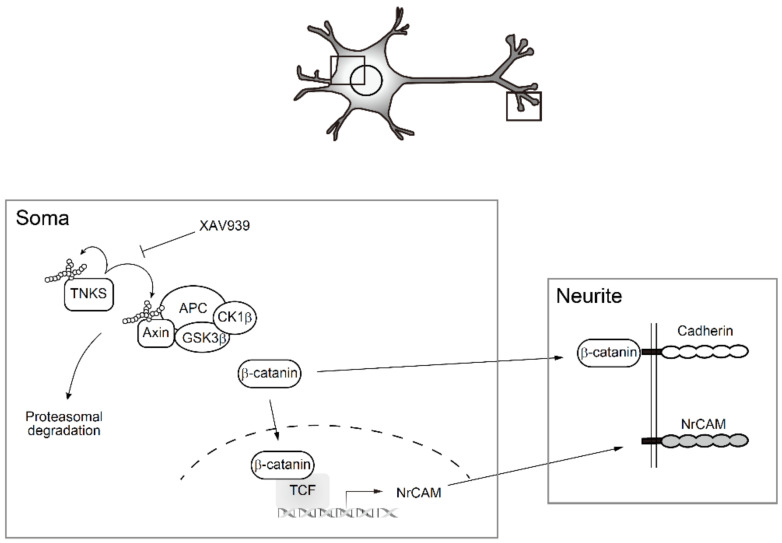
Tankyrase activates β-catenin signaling to enhance neurite outgrowth and synapse formation. TNKS1/2 catalyze poly(ADP-ribosyl)ation of Axin and TNKS1/2 themselves, which activates β catenin signaling. Once translocated to the nucleus, β-catenin promotes transcription of NrCAM gene. β-catenin is also involved in stabilizing cadherin on the cell membrane. TNKS1/2-mediated upregulation of adhesion molecules may enhance neurite outgrowth and synapse formation.

β-catenin also appears to regulate the expression of NrCAM in PC12 cells. The NrCAM promoter contains several binding sites for TCF/LEF, transcription factors required for optimal activation by β-catenin [21,22,23]. NrCAM is mainly expressed in the nervous system and is involved in nerve adhesion and neurite outgrowth [21,22,23]. When an anti-NrCAM antibody was injected into the central canal of the spinal cord of embryonic chicks, commissural axons in the spinal cord failed to extend along the longitudinal axis [22], suggesting that NrCAM plays a key role in axonal guidance. In addition to transcriptional regulation, β-catenin links cadherin to β catenin and cytoskeletal actin to stabilize cell adhesion [37,38]. Because TNKS1/2 are localized in both the soma and neurites, especially at synapses, their activation in neurons may regulate NrCAM transcription in the soma in addition to cadherin stabilization by β-catenin in neurites, both of which are involved in neurite outgrowth and synapse formation (Figure 6).

Inhibition of TNKS1/2 in PC12 cells also suppressed NE uptake, but not NE release. NE uptake is primarily mediated by NET [39]. The fact that XAV939 did not suppress NET expression suggests that it suppresses the subcellular localization and/or activity of NETs. In adipocytes, XAV939 impairs vesicle trafficking and translocation of glucose transporter 4 (GLUT4) to the plasma membrane, leading to suppression of insulin-induced glucose uptake [40]. This suggests that TNKS1/2 regulate vesicle transport and that they are involved in delivering to synapses the proteins necessary for synapse formation/maintenance and synaptic transmission (such as NET).

Although TNKS1/2 positively affect neurite outgrowth and synapse formation, they are themselves targets for autopoly(ADP-ribosyl)ation [6,7], which promotes their ubiquitination-dependent degradation. This suggests TNKS1/2 regulate their own expression levels to finely regulate wnt/β-catenin signaling for neurite outgrowth and synapse formation in neurons.

## 4. Materials and Methods

### 4.1. Reagents

PJ34 was purchased from Enzo Life Sciences (Farmingdale, NY, USA); ABT888 from APExBIO (Houston, TX, USA); XAV939 from Cayman chemical company (Ann Arbor, MI, USA); MG-132, ICG-001, and PNU-74654 from Selleckchem (Houston, TX, USA); nerve growth factor-7S (NGF) from Sigma-Aldrich (Burlington, MA, USA); rabbit polyclonal anti-TNKS1/2 antibody (H-350), mouse monoclonal anti-MAP2 antibody (AP20), mouse monoclonal anti-PAR antibody (10H), and ADP-HPD from Santa Cruz Biotechnology (Dallas, TX, USA); rabbit monoclonal anti-Axin1 antibody (C76H11), and rabbit monoclonal anti-unphosphorylated (Ser33/Thr41) β-catenin antibody (D13A1) from Cell Signaling Technology (Danvers, MA); DAPI, Alexa Fluor 488-conjugated goat anti-rabbit IgG, and Alexa Fluor 568-conjugated goat anti-mouse IgG from Thermo Fisher Scientific (Waltham, MA, USA)); and predesigned primers for real-time PCR from Takara Bio (Kusatsu, Japan).

### 4.2. Cell Culture

Primary cultures of hippocampal neurons were prepared from neonatal (P0) C57BL/6JJ mice using the SUMITOMO Nerve-Cell Culture System (Sumitomo Bakelite, Tokyo, Japan). Hippocampal neurons were then cultured in Neurobasal Medium (Thermo Fisher Scientific) containing 2% B-27 Supplement (Thermo Fisher Scientific) at 37 °C and 5% CO_2_, replacing half the volume of the medium every 5 days.

PC12 rat pheochromocytoma cells were cultured in Dulbecco’s modified Eagle’s medium (DMEM) containing 10% fetal bovine serum (FBS), 10% horse serum (HS), 100 units/mL penicillin, and 100 μg/mL streptomycin at 37 °C and 5% CO_2_ on collagen-coated dishes. For neurite outgrowth, PC12 cells were cultured in DMEM containing 50 ng/mL NGF, 0.5% FBS and 0.1% HS, 100 units/mL penicillin, and 100 μg/mL streptomycin.

### 4.3. Norepinephrine Uptake

PC12 cells were cultured in 6-well plates (2 × 10^5^ cells) at 37 °C and 5% CO_2_. Cells were treated with XAV934 (10 μM) for 18 h and then [^3^H]-norepinephrine (NE) (1 μCi/mL, NET678, PerkinElmer Inc., Waltham, MA, USA) for 4 h. After washing with serum-free DMEM, the labeled cells were treated with A23187 (5 μM) for 10 min to evoke Ca^2+^-induced NE release. The medium was collected and centrifuged (7000× *g*, 2 min, 4 °C), and the [^3^H] content of the supernatant was measured by liquid scintillation (LSC-6100, Aloka, Tokyo, Japan).

### 4.4. Immunocytochemistry

Primary hippocampal neurons and PC12 cells (5 × 10^4^ cells) cultured on glass bottomed dishes were fixed with 4% paraformaldehyde (PFA; 20 min, 4 °C), permeabilized with 0.5% Triton X-100, and blocked with Blocking One (Nacalai Tesque, Kyoto, Japan) (1 h, room temperature). After incubation (overnight, 4 °C) with primary antibodies at an adequate dilution, cells were treated with Alexa fluor 488-conjugated goat anti-rabbit IgG or Alexa fluor 568-conjugated goat anti-mouse IgG (1:500) (1 h, room temperature) then washed three times with PBS and incubated with 300 nM DAPI (10 min, room temperature) to stain the nuclei. Cells were imaged using a confocal microscope (Zeiss LSM 700 Meta; Carl Zeiss, Jena, Germany) equipped with an oil-immersion objective (63×, numerical aperture = 1.4). The optical section thickness was 300 μm to observe all the neurites. Fluorescence data were processed using FijiJ (U.S. National Institutes of Health, Bethesda, MD, USA). Neurite lengths were determined using NeurophologyJ.

### 4.5. Real-Time PCR

PC12 cells were cultured in 12-well plates (2 × 10^5^ cells) at 37 °C and 5% CO_2_. Total mRNA was extracted using Sepazole RNA II Super, and cDNA was synthesized using a S1000 thermal cycler (Bio-rad, Hercules, CA, USA) with a Prime Script RT reagent kit. The PCR products were analyzed with Thermal Cycler Dice (Takara Bio) using SYBR Premix Ex Taq II and predesigned primers.

### 4.6. SDS-PAGE and Western Blotting

PC12 cells (5 × 10^5^ cells) cultured in 6-well plates at 37 °C and 5% CO_2_ were lysed in 50 mM Tris-HCl (pH 7.4) containing 2% SDS. After adjusting the protein concentration in the lysate, samples were prepared with NuPAGE LDS sample buffer (4×, Thermo Fisher Scientific) and NuPAGE sample reducing agent (10×, Thermo Fisher Scientific), electrophoresed on 4–12% NuPAGE bis-tris gel (Thermo Fisher Scientific), and transferred to nitrocellulose membranes (Thermo Fisher Scientific). The membranes were then blocked with Blocking One for 1 h at 37 °C, reacted with primary antibody overnight at 4 °C, washed with TBS containing 0.1% Tween 20, treated with secondary antibody (anti-rabbit or anti-mouse antibody) for 1 h at room temperature, washed again with TBS containing 0.1% Tween 20, and treated with an additional secondary antibody (anti-rabbit or anti-mouse antibody, HRP conjugate, (Promega, Madison, MI, USA)) for 1 h at room temperature. Finally, the membranes were reacted with SuperSignal West (Thermo Fisher Scientific), and chemiluminescence was detected using an Amersham Imager 600.

### 4.7. Pull-Down Assay with GST-Binding Macrodomain

PC12 cells cultured in 6-well plates (5 × 10^5^ cells) at 37 °C and 5% CO_2_ were lysed in buffer containing 50 mM Tris-HCl (pH 7.4), 200 mM NaCl, 1 mM EDTA, 1% Triton X-100, 10% glycerol, 1 mM DTT, 10 μM PJ34, 1 μM ADP-HPD, and protease inhibitor cocktail (Roche, Basel, Switzerland). The resultant lysate was mixed with GST-macrodomain immobilized on Glutathione Sepharose 4B beads (4 h, 4 °C) on a rotating wheel. After washing three times with the buffer, complexes were collected in LDS sample buffer.

### 4.8. Statistical Analysis

Statistical analysis was performed using Sigmaplot (Systat Software Inc., Chicago, IL, USA). Significance was determined using paired *t*-tests or Student’s *t*-test for pairwise comparison or one-way ANOVA with post hoc Dunnett’s test. Data are presented as means ± S.E.M of values from the indicated numbers of experiments. Values of *p* < 0.05 were considered significant. All representative experiments were repeated three times.

## Data Availability

Not applicable.
